# Generation by Reverse Genetics of an Effective, Stable, Live-Attenuated Newcastle Disease Virus Vaccine Based on a Currently Circulating, Highly Virulent Indonesian Strain

**DOI:** 10.1371/journal.pone.0052751

**Published:** 2012-12-21

**Authors:** Sa Xiao, Baibaswata Nayak, Arthur Samuel, Anandan Paldurai, Mallikarjuna Kanabagattebasavarajappa, Teguh Y. Prajitno, Eny E. Bharoto, Peter L. Collins, Siba K. Samal

**Affiliations:** 1 Virginia-Maryland Regional College of Veterinary Medicine, University of Maryland, College Park, Maryland, United States of America; 2 Japfa Comfeed Indonesia, Jakarta, Indonesia; 3 Vaksindo Satwa Nusantara, Jakarta, Indonesia; 4 Laboratory of infectious diseases, National Institute of Allergy and Infectious Diseases, National Institute of Health, Bethesda, Maryland, United States of America; College of Medicine, Hallym University, Republic of Korea

## Abstract

Newcastle disease virus (NDV) can cause severe disease in chickens. Although NDV vaccines exist, there are frequent reports of outbreaks in vaccinated chickens. During 2009–2010, despite intense vaccination, NDV caused major outbreaks among commercial poultry farms in Indonesia. These outbreaks raised concern regarding the protective immunity of current vaccines against circulating virulent strains in Indonesia. In this study, we investigated whether a recombinant attenuated Indonesian NDV strain could provide better protection against prevalent Indonesian viruses. A reverse genetics system for the highly virulent NDV strain Banjarmasin/010/10 (Ban/010) isolated in Indonesia in 2010 was constructed. The Ban/010 virus is classified in genotype VII of class II NDV, which is genetically distinct from the commercial vaccine strains B1 and LaSota, which belong to genotype II, and shares only 89 and 87% amino acid identity for the protective antigens F and HN, respectively. A mutant virus, named Ban/AF, was developed in which the virulent F protein cleavage site motif “RRQKR↓F” was modified to an avirulent motif “GRQGR↓L” by three amino acid substitutions (underlined). The Ban/AF vaccine virus did not produce syncytia or plaques in cell culture, even in the presence of added protease. Pathogenicity tests showed that Ban/AF was completely avirulent. Ban/AF replicated efficiently during 10 consecutive passages in chickens and remained genetically stable. Serological analysis showed that Ban/AF induced higher neutralization and hemagglutination inhibition antibody titers against the prevalent viruses than the commercial vaccines B1 or LaSota. Both Ban/AF and commercial vaccines provided protection against clinical disease and mortality after challenge with virulent NDV strain Ban/010 (genotype VII) or GB Texas (genotype II). However, Ban/AF significantly reduced challenge virus shedding from the vaccinated birds compared to B1 vaccine. These results suggest that Ban/AF can provide better protection than commercial vaccines and is a promising vaccine candidate against NDV strains circulating in Indonesia.

## Introduction

Newcastle disease (ND) is a highly contagious avian disease with worldwide distribution that can cause severe economic losses in poultry industry [1]. The etiologic agent, Newcastle disease virus (NDV), is an enveloped, cytoplasmic virus that is a member of the genus *Avulavirus* in the family *Paramyxoviridae.* The genome of NDV is a nonsegmented, single-stranded, negative-sense RNA that contains six genes encoding a nucleoprotein (N), a phosphoprotein (P), a matrix protein (M), a fusion glycoprotein (F), a hemagglutinin-neuraminidase glycoprotein (HN), a large polymerase protein (L), and an additional protein V that is expressed by RNA editing during synthesis of the P mRNA [2]. The two surface glycoproteins, HN and F, are the viral neutralization antigens and the major protective antigens. The HN protein is responsible for attachment to the host cell and the F protein mediates fusion of the viral envelope with the cell membrane. The F protein is synthesized as an inactive precursor (F0) that is cleaved by host cell protease into two biologically active F1 and F2 subunits that remain linked by a disulfide bond. Cleavage of the F protein is a prerequisite for virus entry and cell-to-cell fusion.

NDV strains are classified as highly virulent (velogenic), intermediate (mesogenic), or avirulent (lentogenic) on the basis of their pathogenicity for chickens (Alexander 1997). The sequence of the F protein cleavage site is a well-characterized, major determinant of NDV pathogenicity in chickens [3], [4]. The F protein of mesogenic and velogenic strains of NDV typically contains a polybasic cleavage site {(R/K)RQ(R/K)R↓F; basic residues are underlined)} that contains the preferred recognition site for furin {RX(K/R)R↓}, which is an intracellular protease present in a wide range of cells and tissues. Consequently, the F protein of these strains can be cleaved in most tissues, conferring the potential for systemic spread. In contrast, avirulent NDV strains typically have basic residues at the −1 and −4 positions in the cleavage site {(G/E)(K/R)Q(G/E)R↓L)} and depend on a secreted protease (or, in cell culture, added trypsin or chicken egg allantoic fluid) for cleavage. This limits the replication of avirulent strains to the respiratory and enteric tracts, where the secreted protease is found. In addition, the residue at the −1 position that immediately follows the cleavage site can affect the efficiency of cleavage: phenylalanine and leucine are typically found in velogenic and lentogenic strains [5], respectively, and the latter has been associated with reduced cleavability of the APMV-1 F protein [6].

NDV has a single serotype. However, genetic and antigenic diversity are recognized for NDV isolates [7]. NDV strains can have genomes of 15186 nucleotides, 15192 nucleotides, or 15198 nucleotides: most strains with a genome size of 15186 nucleotides were isolated before 1960, while most strains that have been isolated recently have genome sizes of 15192 or 15198 nucleotides [8]. Based on genome length and the sequence of the F gene, NDV strains have been classified into two major classes. The class I strains have been isolated mainly from wild birds and are generally avirulent, whereas class II strains have been recovered from wild and domestic birds and include virulent and avirulent strains. Class I and II viruses are further divided into 9 and 11 genotypes, respectively [9]. The early NDV isolates (class II genotype I-IV) have a genome size of 15186 nucleotides, whereas recent NDV isolates (class II genotype V-VII) have a genome size of 15192 nucleotides. Class I strains have a genome size of 15198 nucleotides [8]. Epidemiological studies have revealed that genotypes V, VI and VII of class II NDV strains (i.e., genome size 15192 nucleotides) are the most prevalent genotypes currently circulating worldwide. Of these, genotype VII has been associated with many recent outbreaks in Asia, Africa, Middle East and South America [7].

Lentogenic NDV strains such as Hitcher B1 and LaSota are used worldwide as live vaccines against ND. However, ND continues to be a major disease problem in poultry. Strains B1 and LaSota belong to genotype II in class II and were isolated some 60 years ago, whereas the predominant genotypes currently circulating worldwide are genotypes V, VI and VII. Although the current vaccines offer substantial protection from disease, they do not completely prevent infection or virus shedding, and disease can occur in vaccinated birds. The recent outbreaks of ND in different parts of the world highlight the inadequacy of currently used vaccines. In contrast, recent studies indicated that inactivated vaccines [10], [11], [12], [13], [14] or a live attenuated vaccine [15] developed from currently circulating genotype strains had increased effectiveness.

**Figure 1:**
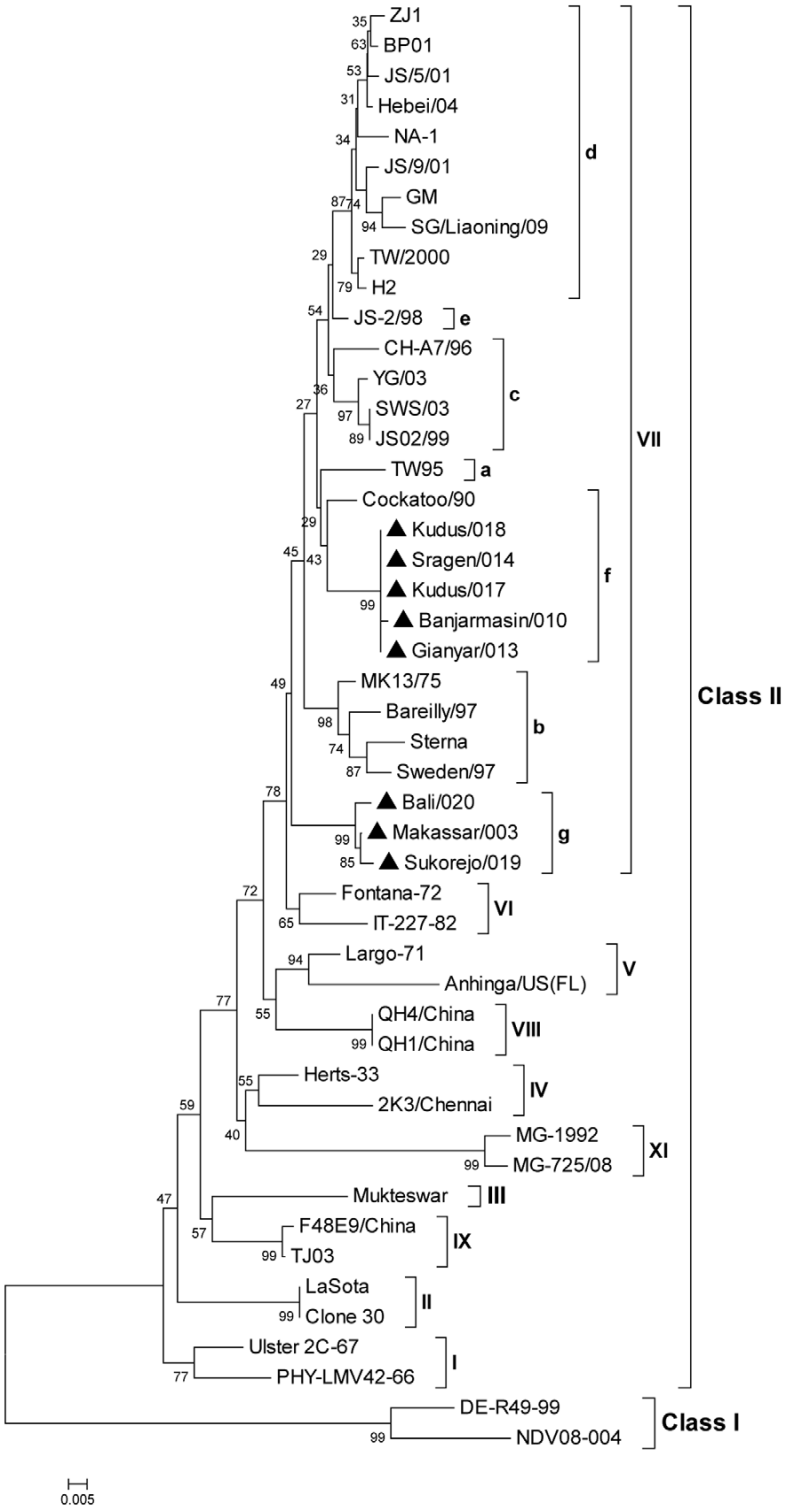
Phylogenetic analysis based on the nucleotide (nt) sequences of complete F open reading frame of Indonesian strains in all genotypes of NDV. The tree was constructed by bootstrap analysis (1000 replications) using the neighbor-joining of the Kimura-2-parameter method for nt differences in the MEGA 4.0 phylogenetic analysis program. Scale bar shows number of base substitutions per site. Bootstrap values are shown at the nodes.

Recently, we have determined complete genome sequences of two NDV strains and the sequence of F and HN genes of six other strains isolated during ND outbreaks in vaccinated commercial chickens in Indonesia during 2009 and 2010 [16] (Table 1). During the outbreaks, mortality was up to 70 to 80% in commercial broilers between 3–4 weeks of age. In laying birds egg production drop up to 30–40% was observed. The genomes of the two Indonesian strains that were completely sequenced are 15192 nucleotides in length. Phylogenetic analysis indicated that the eight Indonesian strains belong to genotype VII in class II, and were further divided into two subgenotypes, VIIf and VIIg (Fig. 1). Analysis of the F gene sequence showed the presence of two types of F protein cleavage site, “RRQKR↓F” and “RRRKR↓F”, which are consistent with the subgenotypes VIIf and VIIg, respectively and also are consistent with a virulent phenotype. The F and HN proteins of the Indonesian strains have 89–90% and 87–88% amino acid sequence identity with F and HN of B1 or LaSota, respectively. In contrast, the amino acid identities of F and HN proteins among these eight Indonesian strains ranged from 96–100% and 94–100%, respectively, and those between B1 and LaSota were 99–100% (Table 2 and 3). These results indicated that sequence divergence exists between circulating and vaccine strains that might be responsible for the incomplete protection of chickens in recent outbreaks in Indonesia. To examine this hypothesis, in the present study we generated a live-attenuated Indonesian strain, Ban/AF, by reverse genetics as a vaccine candidate. This was derived from strain chicken/Banjarmasin/010/10 (Ban/010), one of the two circulating strains noted above that had been sequenced completely, by changing its virulent F protein cleavage site to the avirulent motif.

**Table 1:** Database of NDV Indonesia isolates in this study.

Strain ID	Code	Year of isolation	Geographic area	Sequence		F cleavagesite	Patho-genesis		GenBank accession no.
				Length nt	Genes		MDT	ICPI	
Banjarmasin/010/10	Ban/010	2010	South Kalimantan	15192	Full genes	RRQKR↓F	48	1.88	HQ697254
Gianyar/013/10	Gia/013	2010	Bali	4152	F, HN	RRQKR↓F	60	ND	HQ697257
Sragen/014/10	Sra/014	2010	Central Java	4164	F, HN	RRQKR↓F	51	ND	HQ697258
Kudus/017/10	Kud/017	2010	Central Java	4181	F, HN	RRQKR↓F	54	ND	HQ697259
Kudus/018/10	Kud/018	2010	Central Java	3829	F, HN	RRQKR↓F	57	ND	HQ697260
Sukorejo/019/10	Suk/019	2010	Central Java	15192	Full genes	RRRKR↓F	52	1.88	HQ697255
Makassar/003/09	Mak/003	2009	South Sulawesi	3846	F, HN	RRRKR↓F	57	ND	HQ697256
Bali/020/10	Bal/020	2010	Bali	3835	F, HN	RRRKR↓F	51	ND	HQ697261

ND, not done.

**Table 2:** Identities of amino acid sequences of F protein.

	Gia/013	Sra/014	Kud/017	Kud/018	Suk/019	Mak/003	Bal/020	B1	LaSota
Ban/010	99.8	99.8	99.8	99.8	96.9	96.9	96.0	88.6	89.2
Gia/013		100	100	100	97.1	97.1	96.2	88.6	89.2
Sra/014			100	100	97.1	97.1	96.2	88.6	89.2
Kud/017				100	97.1	97.1	96.2	88.6	89.2
Kud/018					97.1	97.1	96.2	88.6	89.2
Suk/019						100	99.1	89.2	89.7
Mak/003							99.1	89.2	89.7
Bal/020								89.0	89.5
B1									99.5

**Table 3:** Identities of amino acid sequences of HN protein.

	Ban/010	Gia/013	Sra/014	Kud/017	Kud/018	Suk/019	Mak/003	Bal/020	B1
Gia/013	99.8								
Sra/014	100	99.8							
Kud/017	99.8	99.6	99.8						
Kud/018	99.8	99.6	99.8	100					
Suk/019	93.9	93.7	93.9	93.7	93.7				
Mak/003	93.9	93.7	93.9	93.7	93.7	99.6			
Bal/020	93.7	93.5	93.7	93.5	93.5	99.3	99.3		
B1	87.9	87.9	87.9	87.9	87.9	87.9	86.9	86.9	
LaSota	87.9	87.9	87.9	87.9	87.9	86.9	86.9	86.7	99.1

## Materials and Methods

**Cells and Viruses**

The chicken embryo fibroblast DF1 cell line and human epidermoid carcinoma HEp-2 cell line were grown in Dulbecco’s modified Eagle medium (DMEM) containing 10% fetal bovine serum (FBS) and maintained in DMEM with 5% FBS. Bother cell lines were purchased from ATCC (www.atcc.org). In experiments that required supplementation of exogenous protease for cleavage of the F protein, normal chicken egg allantoic fluid was added to a concentration of 10%.

NDV isolate chicken/Banjarmasin/010/10 (Ban/010) was plaque-purified and propagated in 9-day-old embryonated specific pathogen free (SPF) chicken eggs. Infected allantoic fluid was harvested 2 days post-inoculation (dpi). Other seven Indonesian strains were also plaque-purified. Velogenic strain GB Texas was propagated in the same way. The commercial live NDV vaccines B1 and LaSota were purchased from Pfizer, USA and used directly as provided from the manufacturer.

NDV titers in hemagglutination units (HAU) were determined by hemagglutination (HA) assay using 1% chicken red blood cells (RBC) at room temperature. NDV titers in plaque forming units (PFU) were determined by plaque assay was performed in DF1 cells as previous described [17], [18]. Briefly, confluent DF1 cell monolayers were infected with 10-fold dilution of viruses. After 1 h adsorption at 37°C, the cells were washed with PBS for three times, overlaid with 0.8% methylcellulose in 2% FBS DMEM, and observed for plaque production at 7 dpi. The cells was fixed with methanol and stained with 1% crystal violet. NDV titers in 50%-tissue-culture-infectious-dose (TCID50) units were determined as previously described [19]. Briefly, confluent DF1 cell monolayers were infected with 10-fold dilutions of virus as described for the plaque assay and incubated in medium without methylcellulose. At 36 dpi, the cells were fixed with methanol and infected cells were visualized by immunostaining using a rabbit polyclonal antiserum raised against the NDV nucleocapsid (N) protein [20] followed by a secondary antibody and detection by substrate AEC plus chromogen (Dako, USA). The TCID50 titers were calculated by the method of Reed and Muench (Reed, 1938). NDV titers in 50%-chicken-lethal-dose (CLD50) units were determined by infecting 3-week-old chickens with a 10-fold dilution series, and the CLD50 was determined by the method of Reed and Muench [21].

Modified vaccinia virus strain Ankara expressing the T7 RNA polymerase (MVA/T7) was kindly provided by Dr. Bernard Moss (NIAID, NIH) and was propagated in primary chicken embryo fibroblast (CEF) cells.

All virulent virus-related studies were performed in our USDA approved enhanced biosafety level 3 (BSL-3+) facility following the guidelines and approval of IACUC, University of Maryland.

### Reverse Genetic Constructions and Sequencing

**Figure 2:**
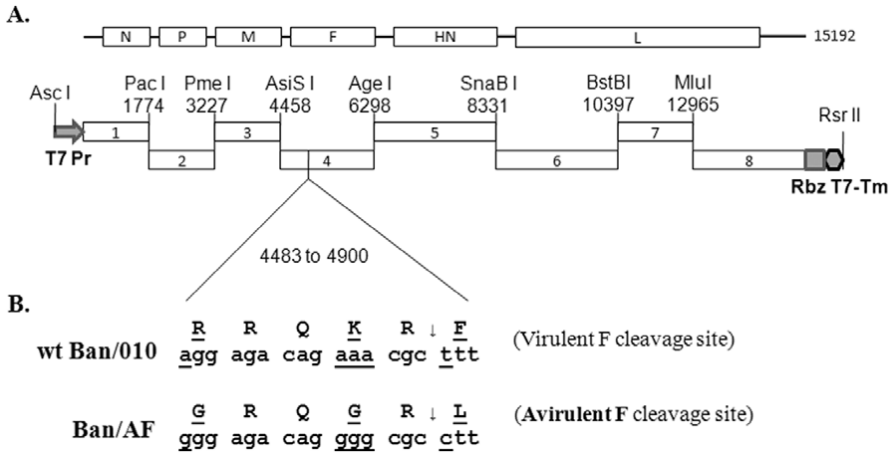
Construction of a full-length antigenomic cDNA of NDV Indonesia strain Ban/010 and modification of its F protein cleavage site. (A) Construction of the antigenomic cDNA. Eight cDNA fragments were generated from viral RNA by RT-PCR using primers that inserted seven unique restriction sites (sequence positions are indicated). Using these sites, the cDNA fragments were inserted sequentially into the pBR322/dr plasmid vector, flanked on the upstream side by a T7 RNA polymerase promoter sequence and on the downstream side by the hepatitis delta virus ribozyme sequence (Rbz) followed by a T7 terminator sequence (Tm). (B) Modification of the F protein cleavage site. The sequence of the cleavage site of the wt Ban/010 virus is shown, as well as the altered cleavage site in mutant Ban/AF. The mutated residues and nucleotides are shown in bold. The arrow indicates where cleavage occurs.

A full-length cDNA of the complete 15,192-nt-long antigenome of NDV Banjarmasin/010/10 (Ban/010) was constructed in plasmid pBR322/dr (19) from eight fragments that were generated by reverse transcription-PCR (RT-PCR) of RNA from NDV-infected embryonated chicken eggs. To facilitate construction, a total of seven restriction sites were introduced into the NDV cDNA during PCR as follows: four sites, namely *Pac I, Pme I, AsiS I* and *Age I*, were created in the downstream untranslated regions (UTR) of the N, P, M and F genes, respectively; a *SnaB I* site was created in the intergenic region between HN and L genes; a *BstB I* site was created in the L ORF without any amino acid changes; and an *Mlu I* site was created to replace the *Pac I* site in the L ORF, resulting in two amino acids changes I1528R and N1529V in region that has considerable amino acid variability among avian paramyxoviruses and may be a region with flexible or disorganized structure. The eight fragments were sequentially cloned into the pBR322/dr plasmid between the T7 promoter and the HDV antigenome ribozyme sequence (Fig. 2A). The F protein cleavage site was mutated using overlapping PCR (Fig. 2B). The mutated F fragment ends were designed with *AsiS I* and *Age I* enzyme sites and was used to replace the corresponding fragment in the full-length cDNA of the pBan/010. The resulting cDNA clone with mutated F gene was named pBan/AF.

Three support plasmids expressing Ban/010 N, P and L proteins were constructed as previous described [22]. Infectious NDV was recovered by transfecting the cDNAs into HEp-2 cells using recombinant MVA vaccinia virus expressing T7 RNA polymerase as previously described [22]. The cell culture medium supernatants were inoculated into embryonated chicken eggs, and successful recovery was identified by allantoic fluid with a positive HA titer. The presence of the created restriction enzyme sites and the sequence of the F protein cleavage site were confirmed by nucleotide sequencing.

For nucleotide sequencing, viral RNA was isolated from allantoic fluid using the RNeasy kit (QIAGEN, USA) according to the manufacturer’s instructions. The first-strand cDNA was synthesized from viral RNA by Superscript II kit (Invitrogen, USA) using random hexamers according to manufacturer’s instructions. PCR was performed using various virus-specific primers and Taq polymerase (Invitrogen, USA). RT-PCR products were sequenced directly. Computer analysis of viral sequences was performed by Lasergene 6. The phylogenetic tree was constructed by MEGA4.0.

### Genetic Stability of the Recombinant Ban/AF Virus

The genetic stability of recombinant Ban/AF virus was examined by three different *in vivo* passage regimens, one involving passage in embryonated chicken eggs, one involving passage in the respiratory tract of 1-day old chicks, and one involving passage in the brains of 1-day-old chicks. For passage in chicken eggs, the virus at a 2^8^ HA titer was diluted 10^5^ fold and inoculated into the allantoic cavity of three 9-day old embryonated SPF chicken eggs. After 3 days incubation at 37°C, the allantoic fluids were examined by HA assay to confirm efficient virus replication, and diluted 10^5^ fold for inoculation into new eggs. After 10 passages, the virus was examined for pathogenicity by MDT and ICPI. The viral genome RNA was isolated and RT-PCR was performed for the sequences of F and HN genes.

For passage in the respiratory tract of 1-day-old chicks, each of three chicks per virus was inoculated with 200 µl of a 2^8^ HAU stock of virus by the oculonasal route by drops administered to both eyes and both nares. Three days after inoculation, the tracheas and lungs were collected into one tube and homogenized in DMEM containing 10X antibiotics (Invitrogen, USA). After centrifugation at 3,000 rpm for 15 minutes, the supernatants were directly inoculated into three new 1-day-old chicks. In addition, at each passage, a separate aliquot of harvested virus was grown in 9-day old embryonated chicken eggs and tested by HA assay to confirm that virus had been recovered from the combined trachea and lung samples. This showed that infectivity was lost during the 4^th^ passage, presumably due to a low titer harvested from passage 3. Therefore the passage 4 inoculation was repeated with passage 3 harvest that had been amplified once in eggs, which was used at a titer of 2^8^ HAU titer. Similarly, virus was not detected following the 7^th^ and 9^th^ passages, and so each of these was repeated using allantoic fluids of the 6^th^ and 8^th^ passaged viruses, respectively. After 10 passages, the virus was examined for pathogenicity by MDT and ICPI. The viral genome RNA was isolated and RT-PCR was performed for the sequences of F and HN genes.

For passage of viruses in brains of 1-day-old chicks, each of three chicks per virus were inoculated with 50 µl of a 2^8^ HA titer virus injected intracerebrally. The brain tissues were collected at 3 days post inoculation (dpi), and homogenized in DMEM. After centrifugation, 50 µl supernatant per bird were inoculated into the brains of three new 1-day-old chicks. After the third passage, the viral genome RNA was isolated and RT-PCR was performed for the sequence analysis.

All experiments involving experimental animals were approved by the committee of IACUC, University of Maryland (protocol number R-09-81) and conducted following the guidelines.

### Replication of the Ban/010, B1, and LaSota Vaccines in 1-day-old and 2-week-old Chickens

The replication of Ban/AF virus in chickens was compared with commercial vaccines B1 and LaSota in 1-day-old chicks and 2-week-old chickens. The lyophilized B1 and LaSota viruses were mixed with the blue-color diluent that was provided with the commercial product. In addition, the Ban/AF virus was diluted in the same blue-color diluent (Pfizer, USA) to the same viral titer of B1 vaccine (10^5.7^ TCID_50_/ml), whereas the LaSota vaccine had a titer of 10^6.1^TCID50/ml. Nine 1-day old chicks per virus and nine 2-week old chickens per virus were inoculated with Ban/AF virus or the live vaccine B1 or LaSota by one drop (∼20 µl) in nasal cavity of each bird. Three birds from each group were sacrificed at 4, 6, and 8 dpi, and tissue samples from five organs (brain, lung, trachea, spleen and pancreas) were collected, and in addition oral and cloaca swabs were collected. The tissue samples were weighted and homogenized in media containing 10 antibiotics, and the swabs also were processed in media containing 10 antibiotics. The supernatants of the samples were tested in 9-day embryonated chicken eggs for HA activity, and titrated in DF1 cells by TCID50 method.

### Immunogenicity of the Ban/010, B1, and LaSota Vaccines in 1-day-old and 2-week-old Chickens

One-day-old chicks and 2-week-old chickens in groups of 3 birds per virus were inoculated with a single nasal drop of the Ban/AF, BI, and LaSota viruses as described above. At 14 dpi, serum samples were collected and analyzed by HI and neutralization assays against each of the three strains: Ban/AF, B1, and LaSota. This study is distinct from replication (above) and challenge (below).

### Challenge Study in 1-day-old and 2-week-old Chickens

The protective efficacy of the Ban/AF vaccine was evaluated in 1-day-old chicks and 2-week-old chickens. One-day-old chicks were inoculated with a single nasal drop of the Ban/AF or BI vaccine as described above or were mock-infected, and 2-week-old chickens were inoculated with a single nasal drop of the Ban/AF and LaSota viruses as described above or were mock-infected. At three weeks post-immunization, the immunized birds were challenged with 100 CLD_50_ of the highly virulent wt strains NDV Ban/010 and GB Texas through the oculonasal route with 200 ul virus per bird in both eyes and both nares. Each challenge group had 10 birds except for the 1-day-old chicks immunized with Ban/AF, which had 12 birds in the Ban/010 challenge group and 11 birds in the GB Texas challenge group. Post-challenge, the chickens were observed daily for 14 days for signs of disease and mortality. Oral and cloacal swabs were collected on days 4 and 7 post-challenge from all chickens, to monitor shedding of the challenge virus. The challenge virus titers in the swab samples were determined by TCID50 assay. Serum samples were collected at day 0, prior to vaccination, at day 21 post-vaccination (i.e., immediately before challenge), and day 14 post-challenge from surviving birds (i.e., the vaccinated birds) and analyzed by HI and neutralization against the eight Indonesian strains (Table 1) and GB Texas.

### Statistical Analysis

Statistically significant differences in serological analysis between different immunized chicken groups were evaluated by one-way analysis of variance (ANOVA). The survival patterns and median survival times were compared by using the log-rank test and chi-square statistics. In the log-rank test, survival curves compare the cumulative probability of survival at any specific time, and the assumption of proportional deaths per time is the same at all time points. Survival data and one-way ANOVA were analyzed with the use of Prism5.0 (Graph Pad Software, Inc., San Diego, CA).

## Results

### Generation of Recombinant wt and F Protein Cleavage Site Mutant Ban/010 Viruses

Eight NDV strains that were isolated during ND outbreaks in commercial chickens in Indonesia during 2009 and 2010 [16] were evaluated for pathogenicity by MDT and ICPI tests (Table 1). This showed that all eight viruses are highly virulent (velogenic) in chickens, consistent with the highly basic nature of their F protein cleavage sites (Table 1). The eight strains were compared for replication in embryonated chicken eggs (not shown), and the strain with the highest titer, chicken/Banjarmasin/010/10 (Ban/010), was selected. The complete sequence of this virus has previously been described [16] (Table 1).

A cDNA clone expressing the antigenome of strain Ban/010 was constructed from eight cDNA segments that were synthesized by RT-PCR from virion-derived genomic RNA (Fig. 1A). The NDV antigenomic cDNA was a faithful copy of the Ban/010 antigenome consensus sequence except for 25 nucleotide changes that were introduced to create seven new unique restriction enzyme sites (*Pac I, Pme I, AsiS I*, *Age I,SnaB I, BstB I* and *Mlu I*) used in the construction (Fig. 2A). This construct contains a T7 promoter that initiates a transcript with three extra G residues at its 5′ end, which increases the efficiency of T7 RNA polymerase transcription and does not interfere with virus recovery. We then made a mutant, called Ban/AF, in which the wt Ban/010 F protein cleavage site (RRQKR↓F, basic residues underlined) was replaced with a cleavage site that is common in avirulent strains including B1 and LaSota: GRQGR↓**L** (the mutated residues are shown in bold) (Fig. 2B). This involved three amino acid substitutions, at the +1, −2, and −5 positions relative to the cleavage site.

The recombinant wt Ban/010 and mutant Ban/AF viruses were recovered by transfection of the respective antigenomic cDNA plasmids and the N, P, and L support plasmids into HEp-2 cells and subsequent inoculation of the cell culture supernatants into embryonated chicken eggs as previous described [22]. The HA positive allantoic fluids was processed to isolate viral genomic RNA, which was subjected to RT-PCR and sequence analysis of the F gene to confirm the sequence of the F protein cleavage site.

### Pathogenicity of the Recombinant Viruses in Embryonated Chicken Eggs and 1-day-old Chicks

The pathogenicity of wt Ban/010, recombinant wt Ban/010, and the F protein cleavage site mutant Ban/AF viruses was evaluated by two standard pathogenicity assays, namely, the mean death time (MDT) test in 9-day-old embryonated SPF chicken eggs and the intracerebral pathogenicity index (ICPI) test in 1-day-old SPF chicks [23]. The MDT value of NDV strains are categorized into velogenic (less than 60 h), mesogeneic (60–90 h), and lentogenic (greater than 120 h) pathotypes. The MDT values of parental Ban/010 and recombinant Ban/010 viruses were 52 and 51 h, respectively. By ICPI test, velogenic strains give values close to 2.0, and lentogenic strains give values close to 0.00. The ICPI values of parental and recombinant Ban/010 viruses were 1.88 (Table 4). These results showed that the recombinant version of the Ban/010 retained the virulent phenotype of its biologically-derived parent virulent, and there was no detectable difference in pathogenicity between the two viruses. However, the MDT value of the mutant Ban/AF virus was greater than 120 h, which is same as vaccine strain B1. The vaccine strain LaSota had a MDT value of 116 h. The ICPI value of the mutant Ban/AF virus was 0.00, which is same as vaccine strain B1, but less than that of vaccine strain LaSota, which had an ICPI value of 0.40 (Table 4). These results suggested that the mutant Ban/AF virus is highly attenuated, similar to vaccine strain B1 and more attenuated than vaccine strain LaSota.

**Table 4:** Pathogenicity and stability of the Ban/AF virus.

	Parental Ban/010	Recombinant Ban/010	Ban/AF			B1	LaSota
			Passage 3in brain	Passage 10in eggs^a^	Passage 10in chicks^b^		
MDT	52	51	>120 h	>120 h	>120 h	>120 h	116
ICPI	1.88	1.88	0.00	0.00	0.00	0.00	0.40

a, Virus was passaged in 1-day-old chicken brain for 3 times. b. Virus was passaged in 9-day-old embryonated chicken eggs for 10 times. c, Virus was passaged through oculonasal route in 1-day-old chicks for 10 times.

### Genetic Stability of the Ban/AF Virus *in vivo*

To examine whether the attenuated Ban/AF virus could revert back to wt virus *in vivo*, the virus was subjected to three different *in vivo* serial passage regimens. In one regimen, virus was serially passaged ten times in 9-day-old embryonated chicken eggs. In the second regimen, virus was passaged ten times in 1-day-old chicks that were infected by the oculonasal route, with virus harvested three days later from the trachea and lungs. This second regimen also included an egg passage following passage 3, 6, and 8 in order to increase the virus titer. In the third regimen, virus was passaged three times in 1-day-old chicks that were infected intracerebrally, with virus harvested three days later from the brain. After the final passage in each regimen, the recovered virus was subjected to RT-PCR and sequence analysis of the F and HN genes. No changes were detected, indicating a lack of reversion or compensatory or adventitious mutations. In addition, each virus was evaluated for MDT and ICPI. These values were found to be unchanged. Thus, the Ban/AF virus was genetically and phenotypically stable during extensive passages *in vivo*.

### Growth Characteristics of the Recombinant Ban/010 and Ban/AF Viruses *in vitro*

**Figure 3:**
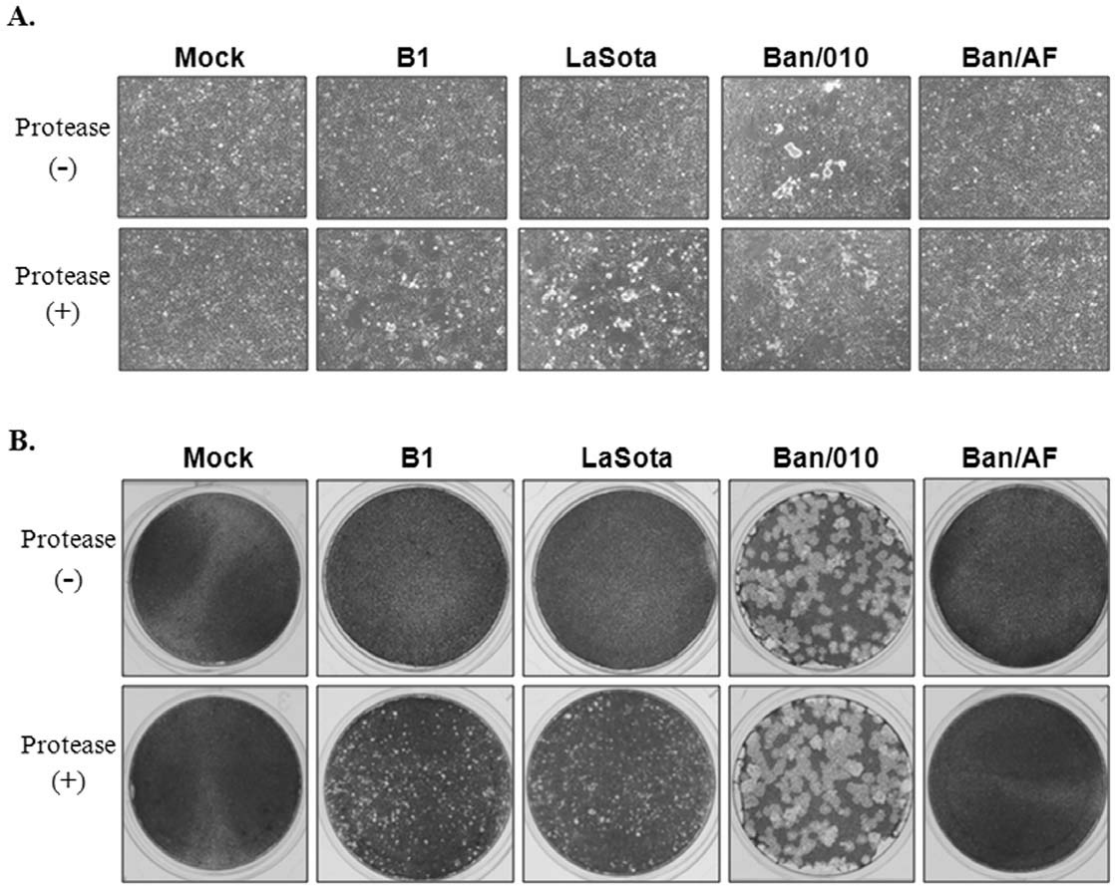
Syncytia and plaque formation by NDV strains in DF1 cells. (A) DF1 monolayers were infected with NDV strains B1, LaSota, Ban/010 and Ban/AF at an MOI of 0.01. The infected cells were cultured in the presence or absence of 10% normal allantoic fluids as exogenous protease. The cells were photographed at 36 hpi. (B) The cells were infected with the indicated viruses at an MOI of 0.01. The infected cells were overlaid by 0.8% methylcellulose DMEM and cultured in the presence or absence of 10% normal allantoic fluid for 7 days. The cells was fixed and stained with 1% crystal violet.

We next examined whether the mutation of the F cleavage site altered the ability of the virus to form syncytia and plaques in cell culture. DF1 cells were infected with Ban/010, Ban/AF, BI, or LaSota viruses at a multiplicity of infection (MOI) of 0.01 in the presence or absence of 10% normal allantoic fluid as protease supplementation. One set of cultures was incubated with fluid overlay to observe the formation of syncytia (Fig. 3A), and another set was incubated under methylcellulose overlay to observe the formation of plaques (Fig. 3B). This showed that the parental Ban/010 virus directed the formation of extensive syncytia and large plaques with or without exogenous protease. The vaccine viruses B1 and LaSota did not cause the formation of syncytia or plaques in the absence of added protease, whereas in the presence of added protease they directed the formation of extensive syncytia and minute plaques. In contrast, the Ban/AF virus did not cause any apparent syncytia (Fig. 3A) or plaques (Fig. 3B) in the presence or absence of exogenous protease.

**Figure 4:**
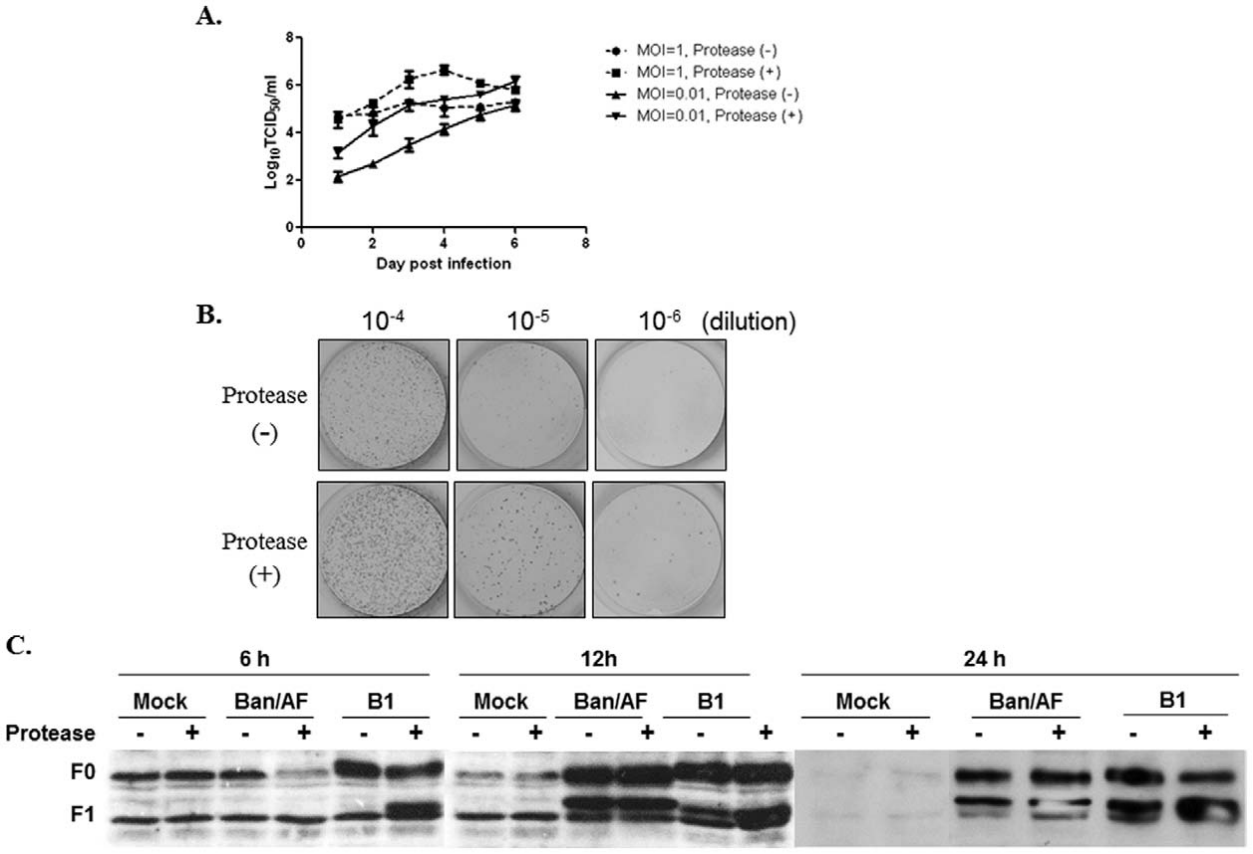
Effect of exogenous protease on the growth and F protein cleavage of the Ban/AF virus in DF1 cells. (A) Cells were infected with the mutant Ban/AF at an MOI of 1 and 0.01, and cultured in the presence or absence of 10% normal allantoic fluid. 100-ul aliquots of culture supernatants were collected daily for titration of viruses by the TCID50 assay. (B) Cells were infected with a 10-fold dilution series of allantoic fluid from Ban/010-infected eggs, and cultured in the presence or absence of 10% normal allantoic fluids. The cells were fixed and immunostained using anti-NDV N protein antiserum (20), followed by incubation with secondary antibody and detection by substrate AEC plus chromogen (Dako, USA). (C) Cleavage of the Ban/AF and B1 F proteins in infected DF1 cells. The cells were infected with viruses at an MOI of 1. The cell lysates were collected at 6, 12 and 24 h post infection. Western-blot was performed with an anti-NDV F cytoplasmic tail peptide rabbit serum.

The growth kinetics of the Ban/AF virus was determined in DF1 cells after infection at an MOI of 1 or 0.01, with or without the presence of exogenous protease (normal allantoic fluid). The culture supernatants were collected daily, and viral titers were analyzed by the TCID50 method (Fig. 4A). With the higher MOI infection (MOI = 1), the titer of virus at day 4 was 10-fold higher in the presence versus the absence of the exogenous protease, although by day 6 the titers were similar. With the lower MOI infection (MOI = 0.01), the titer of virus at day 4 was 100-fold higher the presence versus the absence of exogenous protease, although by day 6 the titers were similar. These results indicated that the protease supplement was able to enhance the replication of Ban/AF virus even if the virus did not form syncytia or plaques. This was further investigated by infecting cells with serial 10-fold dilutions of Ban/AF virus in the presence or absence of added protease. The infected cells were fixed 72 dpi, and virus-infected cells were visualized by immunostaining with anti-NDV N protein antiserum (Fig. 4B). This showed that individual infected cells were readily detected in the presence or absence of added protease, although syncytia were not observed. However, there were 10-fold more infected cells detected in the presence of exogenous protease than in its absence (Fig. 4B). This confirmed that added protease increased the replication of Ban/AF virus even though it did not confer the ability to form syncytia. Further, we examined whether cleavage of the F protein was related to viral replication in presence of exogenous protease by Western-blot analysis with anti-NDV F cytoplasmic tail peptide rabbit serum (Fig. 4C). The results showed that the F protein of Ban/AF virus was cleaved either in the presence or absence of protease after 12 hours infection in DF1 cells. The F protein of B1 virus was effectively cleaved in the presence of protease than in the absence at 6 h and 12 h post infection. There are no significant differences of F protein cleavage in both Ban/AF and B1 virus after 24 h infection (Fig. 4C). However, the Ban/AF replicated to higher titers in the presence of protease than in the absence of protease. We think that in addition to the cleavage site other amino acid sequences of the Ban/AF F protein contribute to the fusion activity.

### Replication, Tissue Tropism and Pathogenicity of the Viruses in 1-day-old and 2-week-old Chickens

**Figure 5:**
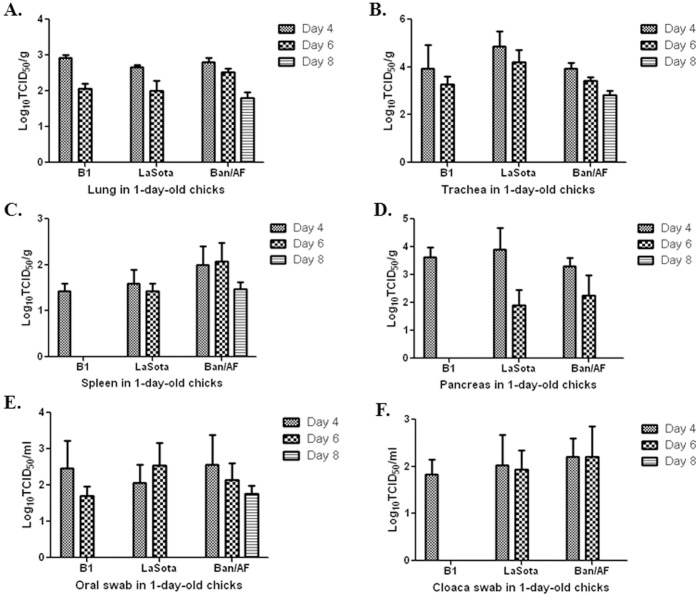
Replication and shedding of vaccine viruses Ban/AF, B1, and LaSota in 1-day old chicks. Chicks in groups of 3 per virus were infected with virus by a single nasal drop each, as described in Materials and Methods. At 4, 6, and 8 dpi, three birds per virus per day were sacrificed, and tissue from the indicated organs, as well as oral and cloaca swabs, were collected. These were assayed for the presence of virus by inoculation into 9-day embryonated chicken eggs, as summarized in Table 2. Samples that were positive in Table 5 were analyzed to determine virus titers by TCID50 assay. Samples: lung (A), trachea (B), spleen (C), pancreas (D), oral swabs (E) and cloaca swabs (F).

We evaluated the replication and pathogenicity of the Ban/AF virus in comparison with vaccine strains B1 and LaSota in 1-day-old chicks. Chicks in groups of ten were inoculated with a single nasal drop (∼20 µL) of Ban/AF (10^5.7^ TCID_50_/ml) or the commercial vaccine B1 (10^5.7^ TCID_50_/ml) or LaSota (10^6.1^TCID50/ml). Three chicks from each group were euthanized on day 4, 6 and 8 dpi and swabs (oral and cloaca) and tissue samples (brain, lung, trachea, spleen, pancreas) were collected. To determine whether virus was present, samples were inoculated into 9-day old embyronated chicken eggs, and 3 days later allantoic fluid was subjected to by HA assay for virus detection (Table 5). Samples that were positive in this egg-infection assay were processed for virus titration by TCID50 assay (Fig. 5). The results showed that, on 4 dpi, all three viruses were detected in all organs and swabs except brain tissue, although detection of all of the viruses in the pancreas was less frequent. On 6 dpi, Ban/AF and LaSota viruses were detected in all samples except the brain, but B1 virus was only found in the lung, trachea and oral swabs. On 8 dpi, only the Ban/AF virus was detected, and was found in the lung, trachea, spleen, and oral swabs (Table 5). Comparison of the viral titers of the samples showed that all three viruses replicated well (>2 log_10_) in the lung, trachea and pancreas (Fig. 5A, 5B and 5D), and somewhat less efficiently in the spleen (Fig. 5C). There were no significant differences in viral titers among days 4, 6, and 8. These data indicated that all three viruses replicated well in 1-day-old chicks, but that the B1 virus was detected somewhat less frequently than the other two viruses. None of the three viruses were detected in the brains of 1-day old chicks following intranasal inoculation.

**Table 5:** vaccine virus replication and shedding in 1-day old chicks.

Post-vaccination	Vaccine	Brian	Lung	Trachea	Spleen	Pancreas	Oral swab	Cloaca swab
Day 4	B1	–	3/3	3/3	2/3	1/3	3/3	3/3
	LaSota	–	2/3	3/3	2/3	1/3	3/3	3/3
	Ban/AF	–	3/3	3/3	3/3	1/3	3/3	1/3
Day 6	B1	–	1/3	1/3	–	–	2/3	–
	LaSota	–	3/3	3/3	3/3	1/3	3/3	1/3
	Ban/AF	–	3/3	3/3	3/3	1/3	3/3	2/3
Day 8	B1	–	–	–	–	–	–	–
	LaSota	–	–	–	–	–	–	–
	Ban/AF	–	2/3	3/3	2/3	–	3/3	–

One-day-old chicks were inoculated intranasally with Ban/AF, B1, or LaSota vaccine virus. Three chicks from each group were euthanized on day 4, 6 and 8 dpi, and oral and cloaca swabs were collected and assayed for infectious virus by inoculation into embryonated chicken eggs. Virus replication was detected by HA assay. The number of virus-positive chicks for each group of three is shown.

**Figure 6:**
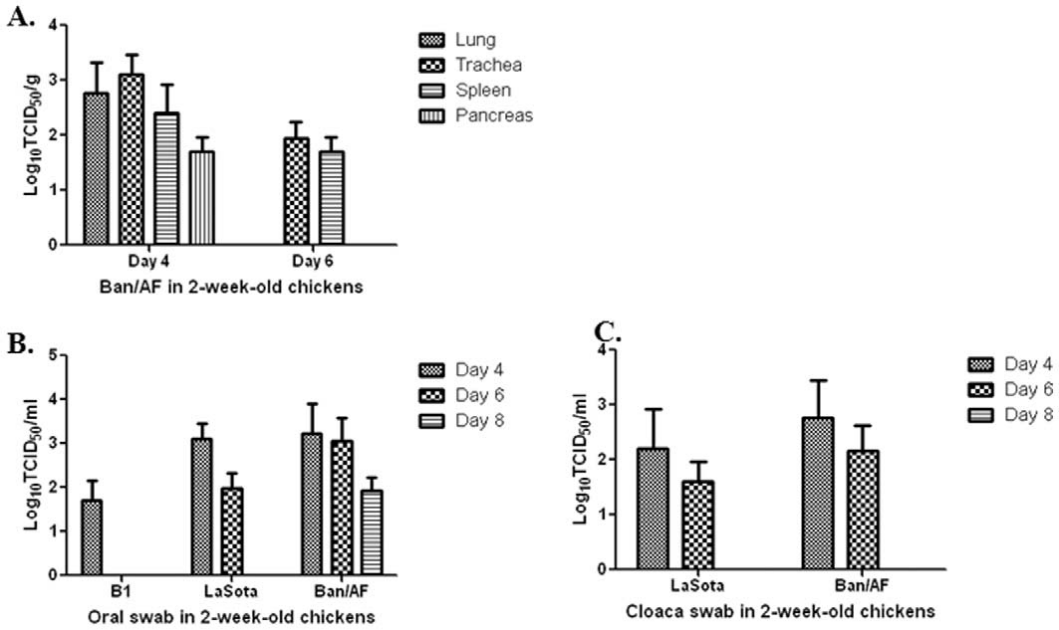
Replication and shedding of vaccine viruses Ban/AF, B1 and LaSota in 2-week-old chickens. Chickens in groups of 3 were infected with virus by a single nasal drop each, as described in Materials and Methods. At 4, 6, and 8 dpi, three birds per virus per day were sacrificed, and tissue from the indicated organs, as well as oral and cloaca swabs, were collected. These were assayed for the presence of virus by inoculation into 9-day embryonated chicken eggs, as summarized in Table 6. Samples that were positive in Table 3 were analyzed to determine virus titers by TCID50 assay. Samples: lung, trachea, spleen, and pancreas (A), oral swabs (B), cloaca swabs (C).

We also evaluated the replication and pathogenicity of these viruses in 2-week-old chickens, in an experiment that was performed in the same way as described above for 1-day-old chicks. In the two-week old chickens, the Ban/AF virus was detected in four organs (lung, trachea, spleen and pancreas) and both swabs (oral, cloaca) at 4 dpi, as well as two organs (lung and spleen) and oral swabs at 6 dpi (Table 6). At 8 dpi, Ban/AF was detected only in nasal swabs (Table 6). B1 and LaSota were not detected in any of the organs at 4, 6 and 8 dpi except for a single isolation of LaSota in the spleen on day 6. The B1 virus was detected only in oral swabs and only at 4 dpi, and the LaSota virus was positive in both oral and cloaca at 4 and 6 dpi. Analysis of the viral titers of the positive tissue samples showed that the Ban/AF virus replicated 10-fold higher in the lung and trachea than in pancreas at 4 dpi (Fig. 6A). The viral titers of the swab samples decreased with post-infection days (Fig. 6B and 6C). These data indicated that the Ban/AF virus replicated more efficiently than the B1 and LaSota vaccine viruses in 2-week-old chickens, and that the replication of all three vaccines was reduced in 2-week-old chickens versus 1-day-old chicks.

**Table 6:** Vaccine virus replication and shedding in 2-week-old chickens.

Post- vaccination	Vaccine	Brian	Lung	Trachea	Spleen	Pancreas	Oral swab	Cloaca swab
Day 4	B1	-	-	-	-	-	2/3	-
	LaSota	-	-	-	-	-	3/3	2/3
	Ban/AF	-	2/3	3/3	3/3	2/3	2/3	1/3
Day 6	B1	-	-	-	-	-	-	-
	LaSota	-	-	-	1/3	-	3/3	1/3
	Ban/AF	-	-	2/3	1/3	-	2/3	-
Day 8	B1	-	-	-	-	-	-	-
	LaSota	-	-	-	-	-	-	-
	Ban/AF	-	-	-	-	-	2/3	-

2-week-old chickens were inoculated intranasally with Ban/AF, B1, or LaSota vaccine virus. Three chicks from each group were euthanized on day 4, 6 and 8 dpi, and oral and cloaca swabs were collected and assayed for infectious virus by inoculation into embryonated chicken eggs. Virus replication was detected by HA assay. The number of virus-positive chicks for each group of three is shown.

### Serological Cross-reaction between Ban/AF and Commercial Vaccine Strains

We investigated the extent of serological cross-reaction between Ban/AF and the commercial vaccine strains B1 and LaSota. One-day-old chicks and 2-week-old chickens were inoculated by a single nasal drop per bird as described above. Serum samples were collected 14 dpi and were analyzed by hemagglutination-inhibition (HI) and neutralization assays against each of the three viruses, Ban/AF, B1, and LaSota. The sera from B1-immunized 1-day-old chicks had the highest HI titer (2^8^ units) to the B1 virus, compared to titers to the LaSota (2^7^ units) and Ban/AF (2^6^ units) viruses. The sera from LaSota-immunized chicks had similar HI titers to LaSota and B1 viruses (2^8^ units), but 2-fold lower HI titer to the Ban/AF virus (2^6^ units). The sera from Ban/AF immunized 1-day old chicks had the same HI titer to Ban/AF and B1 viruses, and somewhat lower to LaSota (data not shown). Similar results for HI serum antibodies were found with 2-week-old immunized chickens. The serum neutralization analysis showed that each serum neutralized the heterologous viruses, but the neutralization titers were always 2 to 4-fold higher to homologous strain than to heterologous strains (data not shown). These results are consistent with the HI results. In general, it was observed that the serum neutralization and HI titers of sera obtained from 1-day-old immunized chicks were 2-fold higher than those obtained from 2-week old immunized chickens.

### Comparison of Protective Efficacy of Ban/AF Versus Commercial Vaccine Strains B1 and LaSota

**Figure 7:**
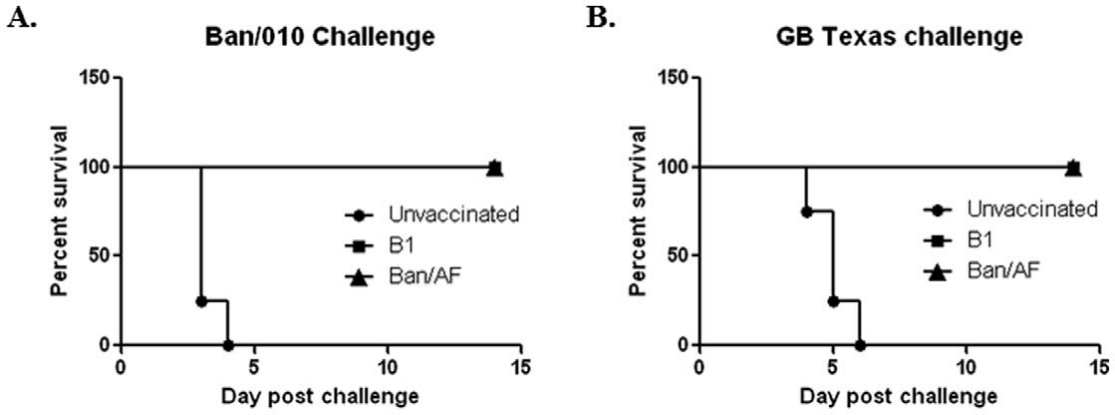
Protective efficacy of vaccine viruses Ban/AF and B1 in 1-day-old chicks against challenge with virulent strains **Ban/010 and GB Texas.** Chicks were inoculated with vaccine viruses Ban/AF or B1 by a single nasal drop each, or were mock-infected, as described in Materials and Methods. At 21 dpi, birds from each group were challenged with 100 LD50 of the Ban/010 or GB Texas viruses by the oculonasal route. The birds were observed daily for survival. Each challenge group had 10 birds except for the 1-day-old chicks immunized with Ban/AF, which had 12 birds in the Ban/010 challenge group and 11 birds in the GB Texas challenge group.

The commercial vaccines B1 and LaSota are usually used for 1-day old chicks and 2-week old chickens, respectively, because the LaSota vaccine can cause mild respiratory disease in 1-day old chicks [24] (although that was not observed in previous experiments in this study in which 1-day-old chicks were inoculated with LaSota virus). Based on this commercial use, the protective immunity of Ban/AF virus was compared with the B1 vaccine in 1-day-old chicks and with the LaSota vaccine in 2-week-old chickens. One-day-old chicks and 2-week-old chickens were inoculated by a single nasal drop per bird as described above, or mock-inoculated. All birds were challenged on day 21 post-vaccination with 100 CLD50 per chicken of the virulent wt Ban/010 (genotype VII, homologous to the Ban/AF vaccine) or GB Texas (genotype II, homologous to the B1 and LaSota vaccines) virus. All Ban/AF- and B1-immunized 1-day-old chicks were fully protected from clinical disease and mortality against Ban/010 (Fig. 7A) or GB Texas (Fig. 7B). Similarly, 2-week-old chickens vaccinated with Ban/AF or LaSota were fully protected from clinical disease and mortality from virulent Ban/010 and GB Texas (data not shown).

**Figure 8:**
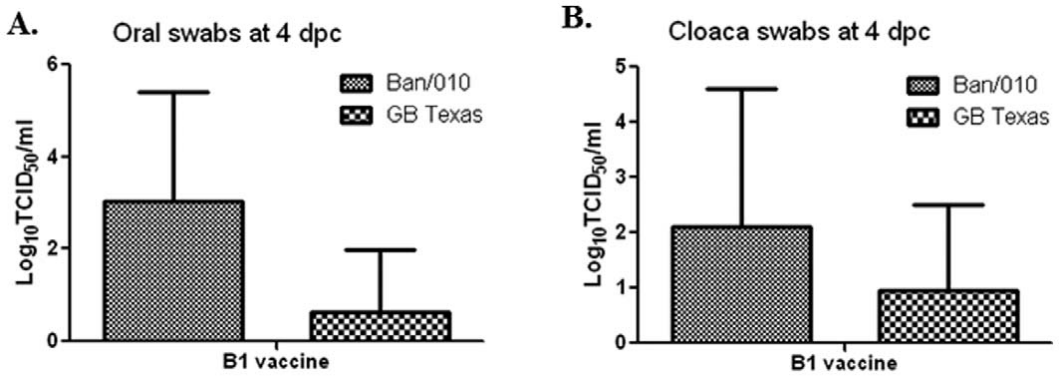
Shedding of Ban/010 or GB Texas challenge virus in 1-day-old chicks previously immunized with Ban/AF or B1. From the experiment shown in Fig. 7, oral and cloaca swabs were collected at 4, 6 and 8 days post-challenge, and samples were assayed for virus detection by inoculation into 9-day embryonated chicken egg. Samples that were positive in Table 7 were titrated by TCID50 assay. Note that virus was detected only in B1-vacciniated chicks.

The shedding of challenge viruses was examined from oral and cloaca swabs on 4 and 7 day post-challenge (dpc) in the 1-day old and 2-week-old birds. In 1-day-old chicks that had been immunized with Ban/AF, no oral or coacal shedding of challenge virus was observed (Table 7). However, in the case of B1-vaccinated 1-day-old chicks, both challenge viruses were detected on day 4 post-challenge, but not on day 7 post-challenge. Analysis of the virus shedding at 4 dpc in B1-immunized 1-day-old chicks showed that the protection against viral shedding by Ban/010 was 30% and 50% in the oral and cloaca, respectively, whereas that against GB Texas was 80% and 70%, respectively (Table 7). The average titers of the challenge Ban/010 virus from the oral and cloacal swabs collected at 4 dpc in B1-immunized 1-day-old chickens were 250-fold and 10-fold higher, respectively, than that of the GB Texas (Fig. 8A and 8B). These data indicated that the Ban/AF vaccine effectively prevented viral shedding in vaccinated 1-day old chickens against both genotype-matched and mismatched virulent viruses, but the B1 vaccine failed to prevent both genotype-matched and mismatched challenge virus shedding. Furthermore, the B1 vaccine was less effective in protecting viral shedding against the heterologous genotype VII Indonesia strain than the homologous genotype II strain GB Texas. In the 2-week-old chickens that were immunized with Ban/AF or LaSota, no challenge virus was detected on either day (day 4 or 7) by inoculation into embryonated eggs or by cell culture methods (data not shown).

**Table 7:** Viral shedding of vaccinated 1-day old birds after challenge.

	Ban/010 virus				GB Texas virus			
	Oral		Cloaca		Oral		Cloaca	
	Day 4	Day 7	Day 4	Day 7	Day 4	Day 7	Day 4	Day 7
B1 vaccine	**7/10**	0/10	**5/10**	0/10	**2/10**	0/10	**3/10**	0/10
Ban/AF	0/12	0/12	0/12	0/12	0/11	0/11	0/11	0/11

### Analysis of Post Challenge Chicken Sera

In the challenge experiment described above, sera were collected prior to vaccination, at 21 days following vaccination (i.e., immediately before challenge), and 14 dpc. All pre-vaccination sera were negative to NDV by the HI test. HI antibodies to NDV were detected in all immunized birds on day 21 post-vaccination. From the experiment in 1-day-old chicks (immunized with Ban/AF or B1), the pre- and post-challenge serum HI antibody titers were determined against the Ban/010 and GB Texas strains. The HI titers of post-challenge sera in all groups were 8- to 32-fold higher than that of pre-challenge sera, but the highest increase in HI titer (32-fold) was observed in the B1-vaccinated group against Ban/010 challenge virus. These results, together with the observed greater breakthrough shedding of Ban/010 challenge virus as noted above, indicated that B1-induced immunity was comparatively less effective in preventing replication of genotype-mismatched challenge Ban/010 virus. The resulting challenge virus breakthrough led to the greater post-challenge antibody response (data not shown). We also analyzed the neutralizing titers of the pre-challenge sera, from the 1-day-old chicks immunized with B1 or Ban/AF, against Ban/010, GB Texas, and the seven other genotype VII virulent Indonesian strains. The serum neutralization results showed that, compared to B1-immunized chicks, Ban/AF-immunized chicks had higher neutralization titers against the seven other Indonesian strains. Similarly, higher neutralization titers were also observed in 2-week-old chicken sera immunized with Ban/AF virus against the seven other Indonesian strains (data not shown). These results suggested that the vaccines Ban/AF, B1 and LaSota were able to induce protective antibodies against GB Texas and Indonesian strains, but the serum neutralization titers were slightly higher against the genotype-matched challenge virus than against genotype-mismatched challenge virus.

## Discussion

Newcastle disease is an economically important disease of poultry, and naturally occurring avirulent NDV strains such as LaSota or B1 are widely used as live attenuated vaccines all over the world. However, outbreaks of velogenic NDV continue to occur in well-vaccinated chickens in different parts of the world, emphasizing the importance for research to understand failures with the current vaccines and to improve vaccine efficacy. The currently used NDV vaccine strains LaSota and B1 were isolated more than 60 years ago in North America and belong to genotype II, whereas, the most commonly circulating velogenic NDV strains around the world at the present time belong to genotype VII [25]. It is thought that the antigenic distance between available vaccine strains versus currently circulating velogenic NDV strains contributes to the vaccine failure in different parts of the world. Therefore, the goal of this study was to determine whether an attenuated, stable, live vaccine virus could be expeditiously generated from a currently circulating virulent virus by reverse genetics, and whether this vaccine could provide better protection against genotype-matched isolates than that provided by the genotype-mismatched vaccine strains LaSota and B1.

In Indonesia, despite routine vaccination of commercial chickens with LaSota and B1 vaccines, outbreaks of virulent NDV is a frequent occurence. During 2009 and 2010, eight virulent NDV strains were isolated from outbreaks in vaccinated chickens in Indonesia. The amino acid sequence identities of the F and HN proteins among these eight strains range from 96 to 100% and 93 to 100%, respectively, compared to 89% and 87% for F and HN between the Indonesian strains and the vaccine strains B1 and LaSota. The genome lengths of Indonesian strains are 1,5192 nucleotides compared to genome lengths of 1,5186 nucleotides for vaccine strains B1 and LaSota. There is 82% nucleotide sequence identity between genomes of the Indonesian isolates and the vaccine strains B1 and LaSota [16]. It’s noteworthy that the sequence divergence between the Indonesian and vaccine strains is similar to the sequence divergence between two subgroups of human respiratory syncytial virus, which share 81% nucleotide sequence identity, or human metapneumovirus, which share 80% nucleotide sequence identity [26], [27]. This suggests that the level of divergence between the circulating Indonesian strains and the vaccine strains currently in use could be an important factor in the poor vaccine protection.

We created a reverse genetics system for NDV strain Ban/010 as the parent for development of an attenuated vaccine strain. Strain Ban/010 was selected because, among the available Indonesian strains, this virus produced the high titer in embryonated chicken eggs, a requirement for large scale vaccine production. Since the amino acid sequence at the F protein cleavage site is a major determinant of NDV virulence [3], [4], the cleavage site of virulent NDV strain Ban/010 was modified into the consensus sequence of avirulent NDV by reverse genetics. As expected, the newly generated recombinant NDV Ban/AF was highly attenuated. This provides another example in which the F protein cleavage site is the major determinant of NDV virulence. To our knowledge, this is the first time report of chicken-origin genotype VII virus developed by a reverse genetics system. A previous study described a genotype VII virus isolated from geese [15].

A major concern was that the mutated F protein cleavage site might revert to a wt or wt-like sequence after passage in chickens. Therefore, we passaged the Ban/AF virus ten times in chicken embryos, or ten times in the respiratory tract of 1-day-old chicks, or three times in the brains of 1-day-old chicks. We then sequenced the F and HN genes and also evaluated viral virulence in MDT and ICPI assays. In each case, there were no sequence changes, and the virus retained its highly attenuated phenotype. Our results differ from those of a previous study in which reversion to virulence was caused by activation of the amino acid sequence of the F0 cleavage site from a non-virulent to a virulent type [28]. Although it is not known why the non-virulent cleavage site was not stable in the other study, one possible reason could be the number of amino acid substitutions required for reversion to virulence. In our study it would require two amino acids for reversion to virulence compared to single-amino acid changes in the other study [29], [30].

It was noteworthy that the Ban/AF virus did not produce syncytia or plaques even in the presence of exogenous protease. On the other hand, the LaSota and B1 strains, which have exactly the same F protein cleavage site sequence as Ban/AF, produced syncytia and small plaques in the presence of exogenous protease. We investigate the efficiency of F protein cleavage in the presence and absence of exogenous protease for Ban/AF and B1 viruses. Our results suggested that sequence other than the cleavage site is necessary for syncytia formation. In any event, the inability of Ban/AF to direct the formation of syncytia or plaques, in contrast to the B1 and LaSota viruses, is indicative of a highly attenuated phenotype. However, when inoculated into 1-day-old and 2-week-old chickens, the Ban/AF virus tended to be detected in harvested tissues and swabs more frequently than the B1 and LaSota strains, although this difference was not great. This suggests that Ban/AF, although highly attenuated, was not severely restricted in tropism.

One-day-old chicks and 2-week-old chickens that were vaccinated either with Ban/AF (genotype VII) or the commercial vaccines LaSota or B1 (genotype II) were fully protected from disease and mortality caused by genotype-matched or -mismatched challenge viruses. These results support the data reported previously by others [10]–[15] that chickens vaccinated with the LaSota strain were fully protected from disease and death following challenge with virulent NDV strains of different genotypes. In addition, both the Ban/AF and the LaSota vaccines prevented viral shedding in 2-week-old chickens following challenge with either genotype-matched or –mismatched virulent virus. In 1-day-old chicks, the situation was somewhat different: the Ban/AF vaccine prevented shedding by both the genotype-matched and –mismatched challenge viruses, whereas shedding was observed in some of the chicks that were vaccinated with the B1 vaccine and challenged with either the genotype-matched or –mismatched virus. This showed that the Ban/AF vaccine was more protective than the standard commercial B1 vaccine in 1-day-old chicks. In addition, in the B1-immunized chicks that shed virus, the titers were less in those that had been challenged with the genotype-matched GB Texas virus than with the mismatched Ban/010 virus. This showed that protection was greater against a homologous- versus heterologous-genotype challenge virus.

Analysis of the serologic response to the vaccination and challenge showed that the vaccinated birds produced higher HI and neutralizing antibody titers against genotype-matched viruses than genotype-mismatched virus. These results support previous reports that showed that the currently available vaccines prevent disease but do not stop viral shedding, whereas genotype-matched vaccines not only prevent disease but also significantly reduce virus shedding [10]–[15]. Another advantage of Ban/AF vaccine is that this virus is highly attenuated which can be used to vaccinate day-old chickens.

In conclusion, this study showed that reverse genetics can be used to rapidly develop a live-attenuated vaccine based on currently circulating virus. The Ban/AF vaccine was stable, was more attenuated than the highly attenuated B1 vaccine, and yet was more protective against homologous and heterologous challenge. Our study also supports the idea that an important factor in the recurrent outbreaks of ND in vaccinated birds is antigenic mismatch between the genotype II commercial vaccine strains and the genotype VII virulent strains that are presently circulating. We found that, although currently available commercial ND vaccines can provide protection against disease, a robust immune response is necessary to prevent viral shedding, which is more effectively induced by a genotype-matched vaccine. However, this was not the sole reason for the superiority of the Ban/AF vaccine, since it completely prevented shedding by both the homolgous- and heterologous-genotype challenge viruses, while vaccination with B1 allowed breakthrough by both. However, it is possible that the protection against disease that was obtained during our laboratory experiments under ideal conditions would be less effective in the field. In addition, it remains to be seen whether a genotype-matched vaccine developed for one geographic area can also be equally effective against virulent strains of the same genotype in other geographic areas. The attenuated Ban/AF virus generated in this study can play a vital role in the control and eradication of ND in Indonesia.
