# Correction: Generation by Reverse Genetics of an Effective, Stable, Live-Attenuated Newcastle Disease Virus Vaccine Based on a Currently Circulating, Highly Virulent Indonesian Strain

**DOI:** 10.1371/journal.pone.0265578

**Published:** 2022-03-14

**Authors:** Sa Xiao, Baibaswata Nayak, Arthur Samuel, Anandan Paldurai, Mallikarjuna Kanabagattebasavarajappa, Teguh Y. Prajitno, Eny E. Bharoto, Peter L. Collins, Siba K. Samal

After this article [1] was published, concerns were raised about vertical discontinuities in Fig 4C, after lanes 6, 12, and 14. The image data underlying Fig 4C are included in S1 File with this notice. The data confirm the results reported in the figure and indicate that the 6 and 12h data were obtained on the same blot whereas the 24h data were obtained on a separate blot. Two lanes (after lane 2) were removed from the 24h blot image when preparing the figure.

This article’s Results section includes six statements that reference data not shown. The results underlying these statements are in S2 File with this notice, and supporting data are in S3 File. For one statement (“In the 2-week old chickens that were immunized with Ban/AF or LaSota, no challenge virus was detected on either day (day 4 or 7) by inoculation into embryonated eggs or by cell culture methods (data not shown).”), the results were negative, i.e. no virus titers were detected per the TCID50 method. The authors noted that graphs of these results would appear blank and so results were not provided in support of this sentence.

The data underlying results reported in this article are available upon request from the first author.

## Supporting information

S1 File Image data to support Fig 4C. (ZIP)

S2 File Results to support ‘data not shown’ statements in [1]. (PPTX)

S3 File Data supporting ‘data not shown’ statements in [1]. (ZIP)
