# Supplementary figures and images for: Correction: Generation by Reverse Genetics of an Effective, Stable, Live-Attenuated Newcastle Disease Virus Vaccine Based on a Currently Circulating, Highly Virulent Indonesian Strain
Source: PLoS One. 2022 Mar 14;17(3):e0265578. doi: 10.1371/journal.pone.0265578 (PMC8920174; doi:10.1371/journal.pone.0265578)

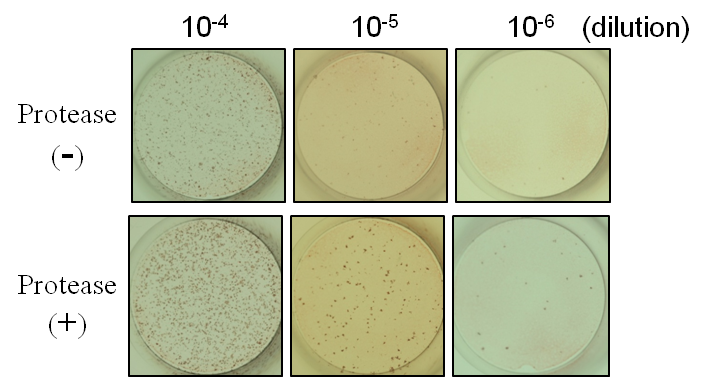

Supplement: S1 File — (ZIP) [file pone.0265578.s001.zip › Original image for Figure 4B.tif]

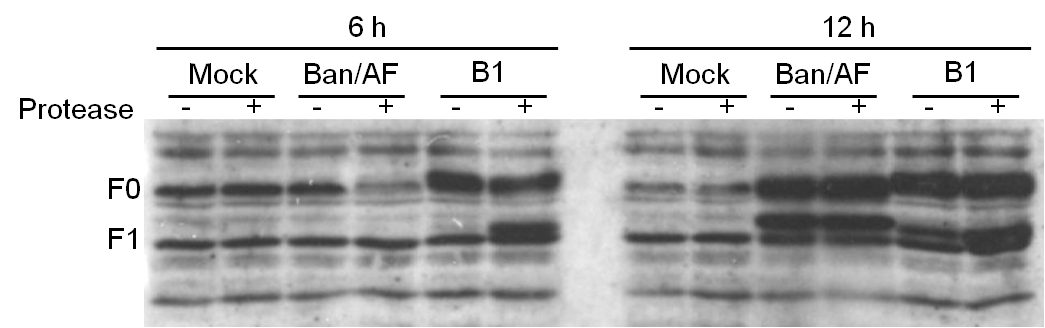

Supplement: S1 File — (ZIP) [file pone.0265578.s001.zip › Original image for Figure 4C (Part 1).tif]

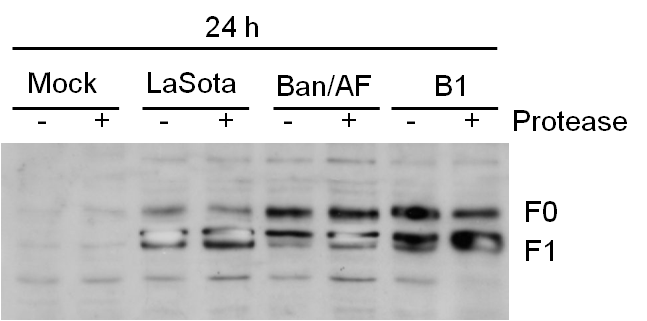

Supplement: S1 File — (ZIP) [file pone.0265578.s001.zip › Original image for Figure 4C (Part 2).tif]
