# Supplementary material for: Correction: Generation by Reverse Genetics of an Effective, Stable, Live-Attenuated Newcastle Disease Virus Vaccine Based on a Currently Circulating, Highly Virulent Indonesian Strain
Source: PLoS One. 2022 Mar 14;17(3):e0265578. doi: 10.1371/journal.pone.0265578 (PMC8920174; doi:10.1371/journal.pone.0265578)
Supplement: S2 File — (PPTX) [file pone.0265578.s002.pptx]

## Slide 1
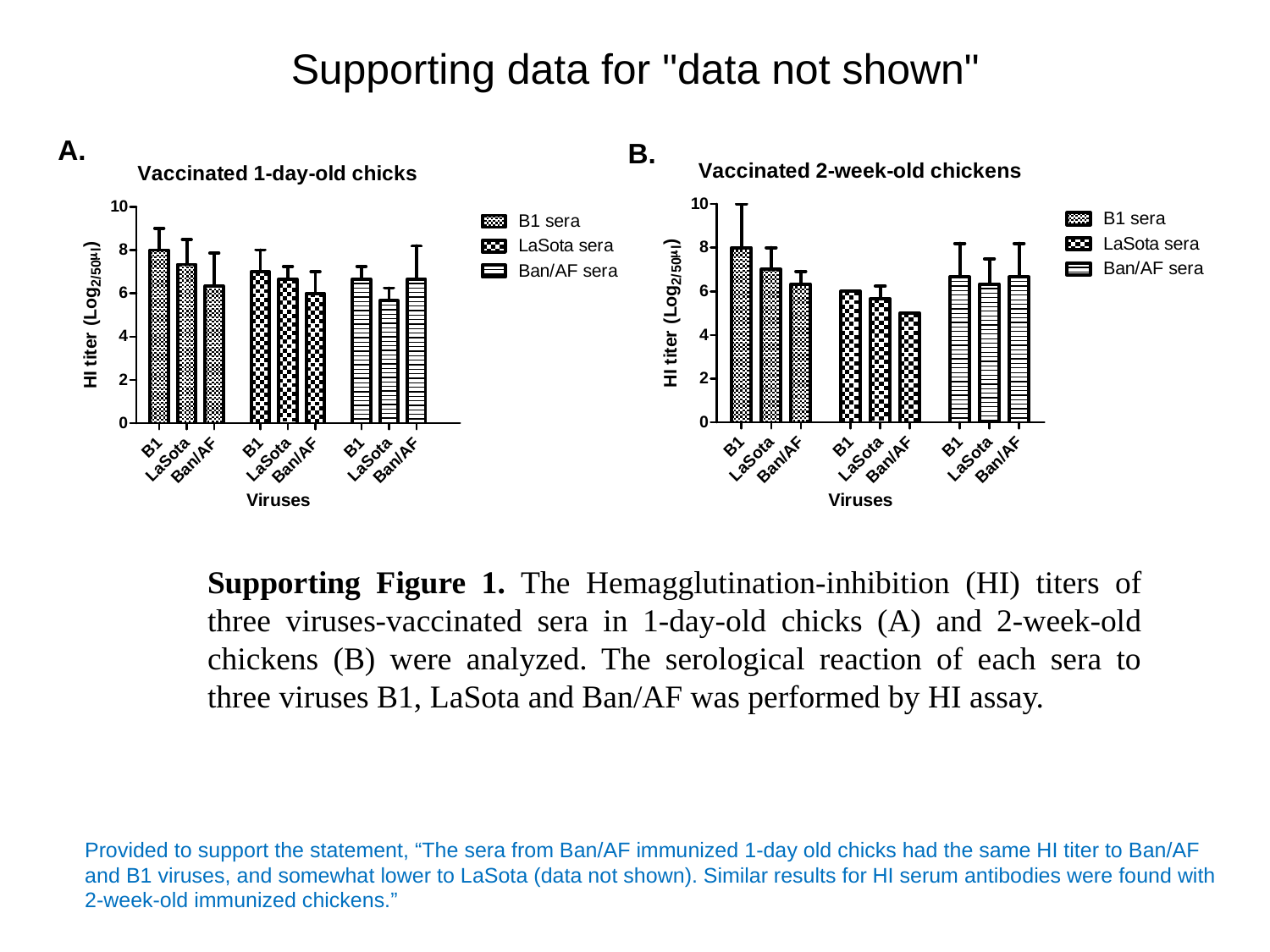

Supporting data for "data not shown"
A.
B.
Supporting Figure 1. The Hemagglutination-inhibition (HI) titers of three viruses-vaccinated sera in 1-day-old chicks (A) and 2-week-old chickens (B) were analyzed. The serological reaction of each sera to three viruses B1, LaSota and Ban/AF was performed by HI assay.
Provided to support the statement, “The sera from Ban/AF immunized 1-day old chicks had the same HI titer to Ban/AF and B1 viruses, and somewhat lower to LaSota (data not shown). Similar results for HI serum antibodies were found with 2-week-old immunized chickens.”

## Slide 2
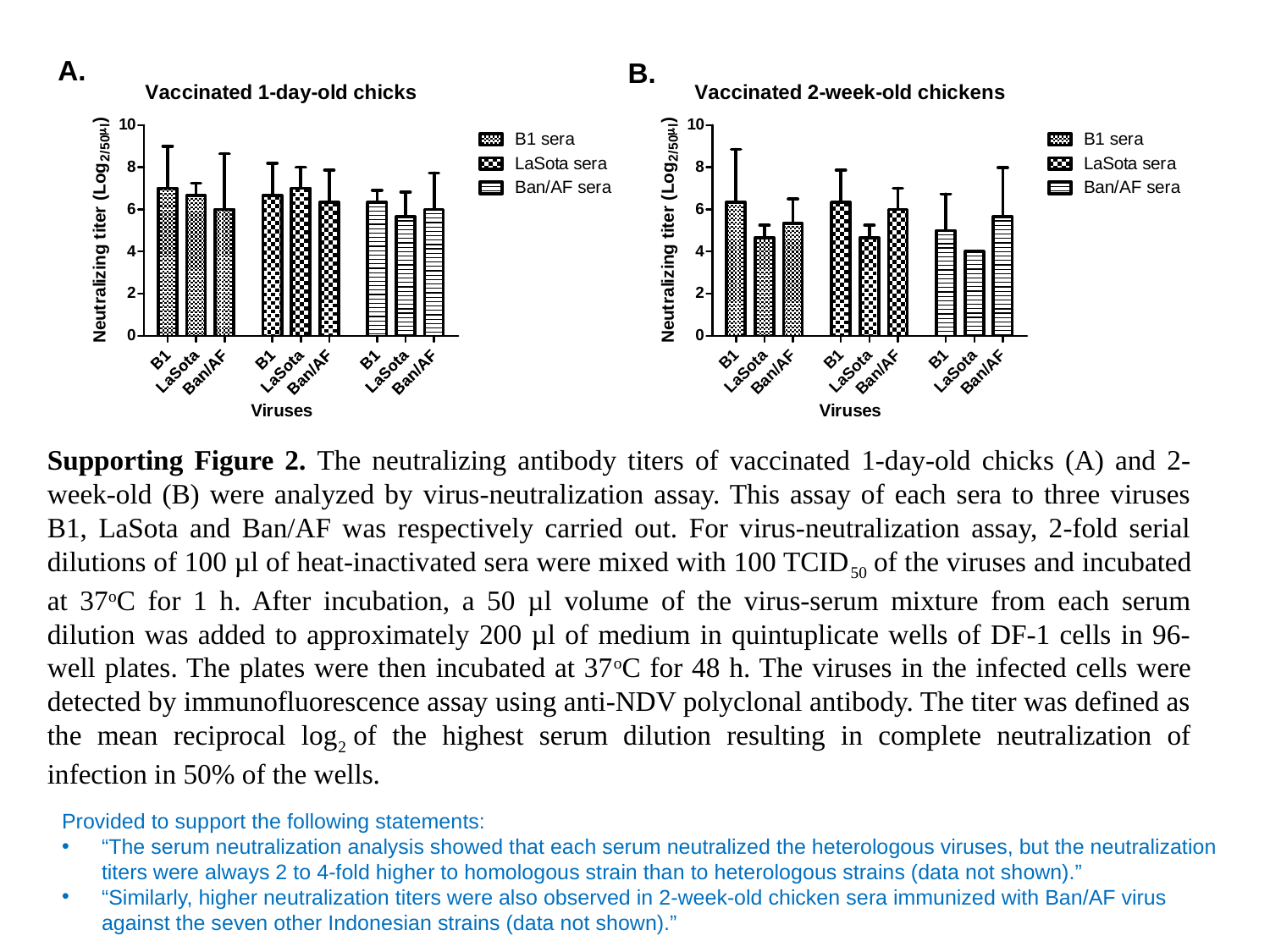

A.
B.
Supporting Figure 2. The neutralizing antibody titers of vaccinated 1-day-old chicks (A) and 2-week-old (B) were analyzed by virus-neutralization assay. This assay of each sera to three viruses B1, LaSota and Ban/AF was respectively carried out. For virus-neutralization assay, 2-fold serial dilutions of 100 µl of heat-inactivated sera were mixed with 100 TCID50 of the viruses and incubated at 37oC for 1 h. After incubation, a 50 µl volume of the virus-serum mixture from each serum dilution was added to approximately 200 µl of medium in quintuplicate wells of DF-1 cells in 96-well plates. The plates were then incubated at 37oC for 48 h. The viruses in the infected cells were detected by immunofluorescence assay using anti-NDV polyclonal antibody. The titer was defined as the mean reciprocal log2 of the highest serum dilution resulting in complete neutralization of infection in 50% of the wells.
Provided to support the following statements:
“The serum neutralization analysis showed that each serum neutralized the heterologous viruses, but the neutralization titers were always 2 to 4-fold higher to homologous strain than to heterologous strains (data not shown).”
“Similarly, higher neutralization titers were also observed in 2-week-old chicken sera immunized with Ban/AF virus against the seven other Indonesian strains (data not shown).”

## Slide 3
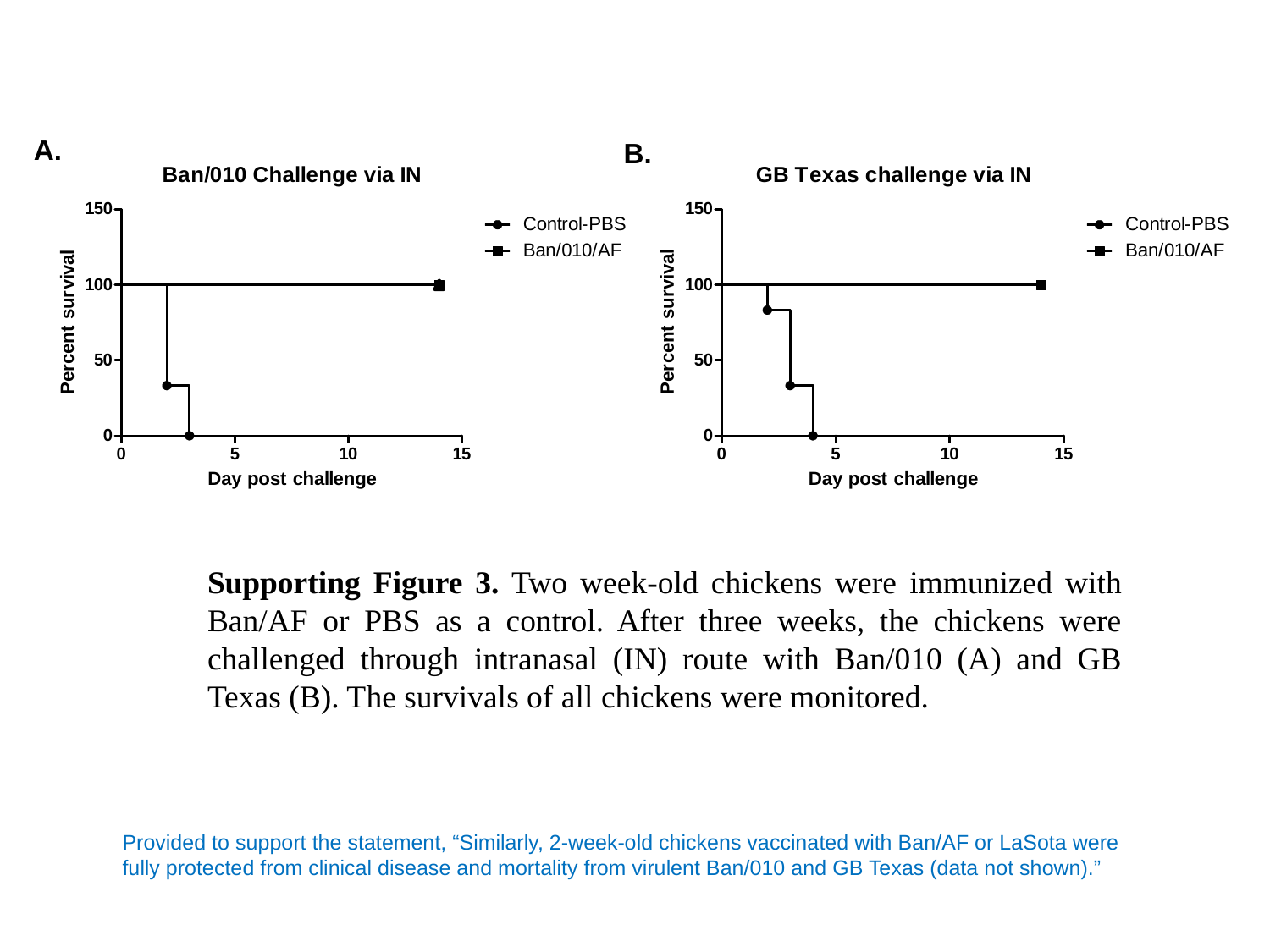

A.
B.
Supporting Figure 3. Two week-old chickens were immunized with Ban/AF or PBS as a control. After three weeks, the chickens were challenged through intranasal (IN) route with Ban/010 (A) and GB Texas (B). The survivals of all chickens were monitored.
Provided to support the statement, “Similarly, 2-week-old chickens vaccinated with Ban/AF or LaSota were fully protected from clinical disease and mortality from virulent Ban/010 and GB Texas (data not shown).”

## Slide 4
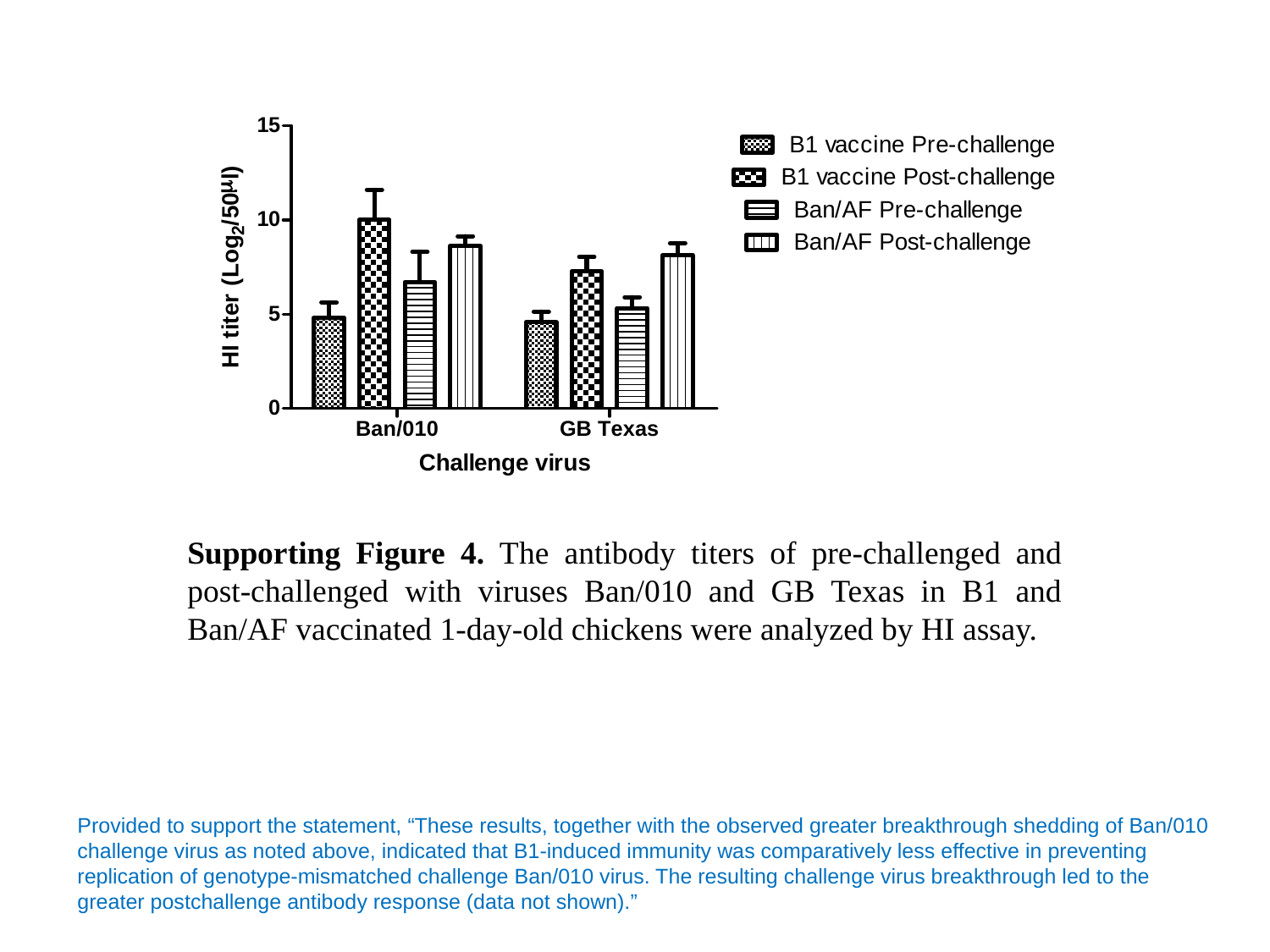

Supporting Figure 4. The antibody titers of pre-challenged and post-challenged with viruses Ban/010 and GB Texas in B1 and Ban/AF vaccinated 1-day-old chickens were analyzed by HI assay.
Provided to support the statement, “These results, together with the observed greater breakthrough shedding of Ban/010 challenge virus as noted above, indicated that B1-induced immunity was comparatively less effective in preventing replication of genotype-mismatched challenge Ban/010 virus. The resulting challenge virus breakthrough led to the greater postchallenge antibody response (data not shown).”
